# Supplementary material for: The role of geomorphic zonation in long-term changes in coral-community structure on a Caribbean fringing reef
Source: PeerJ. 2020 Oct 22;8:e10103. doi: 10.7717/peerj.10103 (PMC7585725; doi:10.7717/peerj.10103)
Supplement: Supplemental Information 1 — (A) Coral colony maximum diameters densities by morphologies and accretion impact sorted by geomorphic zone at Punta Maroma reef in 1985 and 2019. Bran: Branching, Digi: Digitate or digitate, Foli: Foliaceus or platy, Mass: massive, submass_enc: submassive or encrusting forms; RF: Reef front or accretionary zone, HG: Coral hard ground or non-accretionary zone, (B) Coral colony densities by morphologies and by geomorphic zone at Punta Maroma reef in 2019. Bran: Branching, Digi: Digitate or digitiformes, Foli: Foliaceus or platy, Mass: massive, submass_enc: submassive or encrusting forms; RF: Reef front or accretionary zone, HG: Coral hard ground or non-accretionary zone. [file peerj-08-10103-s001.rtf]

	1985	2019	
	key reef-building_
spp.	N/A			key reef-building _spp.	N/A		
Spp.	RF	CG	RF	CG	Av.1985		CG	RF	CG	RF	Av.2019	
AAGA			32.70	14.78	18.70				15.34	18.83	16.26	
ACER	40.73	24.38			56.19		27.00	19.62			21.67	
AFRA									11.50	9.00	10.25	
AHUM										13.33	13.33	
ALAM										10.00	10.00	
APAL	118.59				71.93			90.00			90.00	
APRO								220.00			220.00	
ATEN			47.71		49.98				28.07	21.66	24.01	
CNAT							90.00	20.00			66.67	
DLAB		37.68			37.68		38.00				38.00	
DSTO				11.64	11.64				12.75	15.00	13.20	
FFRA										5.00	5.00	
HCUC									17.00		17.00	
IRIG			13.30	16.63	14.96				11.67		11.67	
MCAV		27.23			26.75		25.10	9.00	35.00		24.99	
MLAM									20.00		20.00	
MMEA				15.52	15.52				39.00	10.00	29.33	
OANN	53.20*	54.41*			45.65*		18.00				18.00	
OFAV							47.30	18.00			45.90	
PAST			27.71	11.88	18.68				12.23	16.81	15.78	
PDIV									8.00	6.67	7.00	
PFUR									30.00	13.17	15.57	
PPOR				13.30	30.59				29.46	17.23	23.90	
PSTR							27.94	19.00			26.79	
SBOU			19.95		19.95				11.25	8.00	10.60	
SINT				6.65	6.65				11.53	18.43	14.86	
SRAD			51.54	6.65	31.59				5.00	10.56	10.00	
SSID	63.18	19.95			26.60		16.43	20.01			17.73	
DCLI				38.24	31.92							
DCYL				39.90	39.90							
DSTR		21.06			21.06							
Average	82.60	32.25	39.47	16.93	42.05		23.93	22.43	16.82	17.19	18.60	

(A)	Coral colony maximum diameters densities by morphologies and accretion impact sorted by geomorphic zone at Punta Maroma reef in 1985 and 2019; RF: Reef front or accretionary zone, CG: Coral- ground or non-accretionary zone. Species Codex: APAL:Acropora palmata,ACER:A.cervicornis, APRO:A. prolifera, AAGA:Agaricia agaricites, AFRA:A. fragilis, AHUM:A. humilis,ALAM:A. lamarcki, ATEN:A. tenuifolia, CNAT:Colpophyllia natans, DSTO:Dichocoenia stokesii,DLAB:Diploria labyrinthiformis, FFRA:Favia fragum, IRIG:Isophyllia rigida, LCUC:Leptoseriscucullata, MMEA:Meandrina meandrites, MCAV:Montastraea cavernosa, MANN CX:M. annularisspp. Complex (Orbicella faveolata,O. annularis), MLAM:Mycetophyllia lamarckiana, PAST:Poritesastreoides, PFUR:P. furcata, PDIV:P. divaricata, PPOR:P. porites, PSTR:Pseudodiploria. strigosa,SSID:Siderastrea siderea, SRAD:S. radians, SBOU:Solenastrea bournoni, SINT:Stephanocoenia intersepta;
   (*)   Diameters are referred to Montrastrea annularis spp. complex. 
(B)	


(B). Coral colony densities by morphologies and by geomorphic zone at Punta Maroma reef in 2019. Bran: Branching, Digi: Digitate or digitiformes, Foli: Foliaceus or platy, Mass: massive, submass_enc: submassive or encrusting forms; RF: Reef front or accretionary zone, HG= CG: Coral- ground or non-accretionary zone. 
