# Supplementary figures and images for: The role of geomorphic zonation in long-term changes in coral-community structure on a Caribbean fringing reef
Source: PeerJ. 2020 Oct 22;8:e10103. doi: 10.7717/peerj.10103 (PMC7585725; doi:10.7717/peerj.10103)

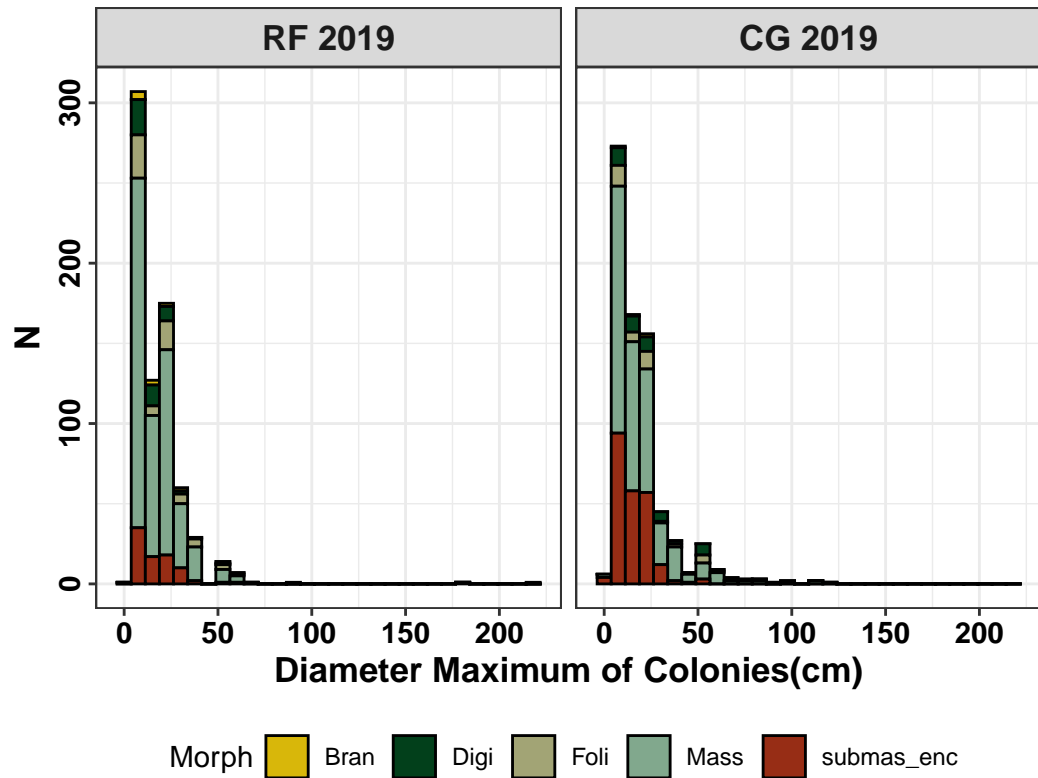

Supplement: Supplemental Information 2 [file peerj-08-10103-s002.pdf]
