# Supplementary material for: The role of geomorphic zonation in long-term changes in coral-community structure on a Caribbean fringing reef
Source: PeerJ. 2020 Oct 22;8:e10103. doi: 10.7717/peerj.10103 (PMC7585725; doi:10.7717/peerj.10103)
Supplement: Supplemental Information 3 — Results of Similarity Percentages test within the sampling group by average abundance of discriminating species by their contribution to the similarity between groups, and the cumulative total (%) of contributions by zones: before the 1990s (RF before 1990s: A1, HG before 1990s: A2), sampling groups by zones for 2019 ( RF 2019: B1, HG 2019: B2) and a comparison ( Dissimilarities) between the two groups in every period (RF and HG before 1990s: C1, RF and HG 2019: C2). RF: Reef front or accretionary zone; HG: Coral hard ground or non-accretionary zone. Av.Abund: Average Abundance; Contrib%: spp. contribution in percentages; Cum.%: cumulative total (%) of contributions (70% cut-off); Av. Diss: average dissimilarities. SD: standard deviation of data. [file peerj-08-10103-s003.rtf]

S2 Data. SIMPER analysis for studies. Results of Similarity Percentages test within the sampling group by average abundance of discriminating species by their contribution to the similarity between groups, and the cumulative total (%) of contributions by zones: before the 1990s (RF before 1990s: A1, CG before 1990s: A2), sampling groups by zones for 2019 ( RF 2019: B1, CG 2019: B2) and a comparison ( Dissimilarities) between the two groups in every period (RF and CG before 1990s: C1, RF and CG 2019: C2). RF: Reef front or accretionary zone; CG: Coral- ground or non-accretionary zone. Av.Abund: Average Abundance; Contrib%: spp. contribution in percentages; Cum.%: cumulative total (%) of contributions (70% cut-off); Av.Diss: average dissimilarities. SD: standard deviation of data    	        	     

One-Way Analysis
Data worksheet
Name: Square Root Transformed Data    

Data type: Abundance
Sample selection: All
Variable selection: All
Parameters
Resemblance: S17 Bray-Curtis similarity
Cut off for low contributions: 70.00%
Factor Groups
Sample	Zone 

A1_Group RF Before 1990s
Average similarity: 52.8

Species	Av.Abund	Av.Sim	Sim/SD	Contrib%	Cum.%	
APAL	    2.60	 16.80	  1.77	   31.79	31.79	
ACER	    1.77	 12.98	  1.59	   24.57	56.36	
ATEN	    1.92	  8.05	  1.05	   15.24	71.61	

A2_Group CG Before 1990s
Average similarity: 42.57

Species	Av.Abund	Av.Sim	Sim/SD	Contrib%	Cum.%	
MCAV	    2.00	 13.96	  1.55	   32.79	32.79	
DSTO	    0.73	  5.65	  1.50	   13.27	46.06	
SSID	    0.93	  5.08	  1.15	   11.93	58.00	
MANN CX	    1.18	  3.56	  0.56	    8.36	66.36	
AAGA	    0.74	  3.44	  0.81	    8.07	74.43	

	
B1_Group CF 2019
Average similarity: 58.35

Species 	Av.Abund	Av.Sim	Sim/SD	Contrib%	Cum.%	
PAST	    6.73	 24.83	  3.14	   42.56	42.56	
AAGA	    3.45	 10.33	  1.80	   17.70	60.26	
SSID	    3.16	 10.29	  3.21	   17.63	77.89	

B2_Group CG 2019
Average similarity: 70.7

Species	Av.Abund	Av.Sim	Sim/SD	Contrib%	Cum.%	
AAGA	    5.65	 18.27	  3.50	   25.84	25.84	
SSID	    3.95	 12.33	  4.71	   17.43	43.27	
PAST	    3.83	 12.17	  4.16	   17.22	60.49	
MCAV	    3.16	  9.45	  2.85	   13.36	73.86	

C1_Groups RF & CG Before 1990s
Average dissimilarity = 77.7

Species	Av.Abund.RF	Av.AbundHG	Av.Diss	Diss/SD	Contrib%	Cum.%	
APAL	    2.60	    0.04	  12.19	   2.28	   15.69	15.69	
MCAV	    0.15	    2.00	   9.16	   1.94	   11.78	27.47	
ATEN	    1.92	    0.00	   8.78	   1.23	   11.29	38.76	
ACER	    1.77	    0.66	   6.93	   1.39	    8.92	47.69	
MANN CX	    0.92	    1.18	   5.95	   1.21	    7.65	55.34	
SSID	    0.51	    0.93	   4.16	   1.24	    5.35	60.69	
PAST	    0.81	    0.58	   3.98	   1.25	    5.13	65.81	
DSTO	    0.00	    0.73	   3.68	   1.94	    4.74	70.55	
APAL	    2.60	    0.04	  12.19	   2.28	   15.69	15.69	
MCAV	    0.15	    2.00	   9.16	   1.94	   11.78	27.47	


C2_Groups RF & CG 2019
Average dissimilarity = 47.41
Group _HG 2019	Group _RF 2019

Species	Av.Abund.RF	  Av.Abund.HG	Av.Diss	Diss/SD	Contrib%	Cum.%	
PAST	    3.83	    6.73	   6.14	   1.54	   12.96	12.96	
MCAV	    3.16	    0.30	   5.76	   2.32	   12.15	25.11	
AAGA	    5.65	    3.45	   5.14	   1.30	   10.84	35.95	
ATEN	    0.79	    2.09	   4.09	   1.29	    8.62	44.57	
PSTR	    2.11	    0.57	   3.35	   1.76	    7.07	51.64	
PPOR	    2.27	    2.12	   3.20	   1.30	    6.74	58.38	
SSID	    3.95	    3.16	   2.89	   1.43	    6.10	64.48	

APAL:Acropora palmata,ACER:A.cervicornis, APRO:A. prolifera, AAGA:Agaricia agaricites, AFRA:A. fragilis, AHUM:A. humilis,ALAM:A. lamarcki, ATEN:A. tenuifolia, CNAT:Colpophyllia natans, DSTO:Dichocoenia stokesii,DLAB:Diploria labyrinthiformis, FFRA:Favia fragum, IRIG:Isophyllia rigida, LCUC:Leptoseriscucullata, MMEA:Meandrina meandrites, MCAV:Montastraea cavernosa, MANN CX:M. annularisspp. Complex (Orbicella faveolata,O. annularis), MLAM:Mycetophyllia lamarckiana, PAST:Poritesastreoides, PFUR:P. furcata, PDIV:P. divaricata, PPOR:P. porites, PSTR:Pseudodiploria. strigosa,SSID:Siderastrea siderea, SRAD:S. radians, SBOU:Solenastrea bournoni, SINT:Stephanocoenia intersepta
