# Supplementary material for: The role of geomorphic zonation in long-term changes in coral-community structure on a Caribbean fringing reef
Source: PeerJ. 2020 Oct 22;8:e10103. doi: 10.7717/peerj.10103 (PMC7585725; doi:10.7717/peerj.10103)
Supplement: Supplemental Information 5 [file peerj-08-10103-s005.rtf]

A.	Beta Diversity Changes in coral communities at Punta Maroma. Pair-wise comparisons of Distance-based test for homogeneity of multivariate dispersions (PERMDISP) on Jaccard Similarity matrix as a measurement of changes in beta diversity after transforming Abundance data to presence /absence. RF: Reef front or accretionary zone; CG: Coral- ground or non-accretionary zone in both periods of time (before the 1990s and 2019). P(perm): P-values obtained using permutations. SE: standard error of means listed by average for each group. 


Name: Jaccard matrix
Data type: Similarity
Selection: All
Transform: Presence/absence
Resemblance: S7 Jaccard

Group factor: Temporal
Number of permutations: 9999
Number of groups: 4
Number of samples: 38


DEVIATIONS FROM CENTROID
F: 4.8323  df1: 3   df2: 34
P(perm): 0.016

PERMDISP JACCARD PAIRWISE COMPARISONS
Groups	      t	P(perm)	
(RF_before 1990,CG_before 1990)	  1.863	 0.103	
(RF_before 1990,CG_2019)	  1.549	 0.153	
(RF_before 1990,RF_2019)	  0.963	 0.395	
(CG_before 1990,CG_2019)	  4.594	 0.0004	
(CG_before 1990,RF_2019)	  0.924	 0.4109	
(CG_2019,RF_2019)	  2.975	 0.0091	

MEANS AND STANDARD ERRORS
Group	Size	Average	    SE	
RF_before 1990	   9	 34.605	3.5082	
CG_before 1990	   9	 42.723	2.5849	
CG_2019	  10	 28.751	1.7118	
RF_2019	  10	 39.025	2.9985	

B.	Results of two-way permutational MANOVA (PERMANOVA) with two contrast analyses for year-factor and pair-wise tests for PERMANOVA of zone in time term (Year x Zone) for pair of levels of factor Zone. RF: Reef front or accretionary zone; CG: Coral- ground or non-accretionary zone in both periods of time (before the 1990s and 2019). P(perm): P-values obtained using permutations. P(MC): Monte Carlo p-values. Data: Bray _Curtis similarities matrix of square root transformed spatial living coverage 2019 standardized by Total and permutation of residuals under reduced model with 9999 permutations  

PERMANOVA PAIRWISE COMPARISONS
Source	df	SS	MS	Pseudo-F	P(perm)	perms	P(MC)	
Year**	2	28855	 14428	  13.771	 0.0001	  9919	0.0001	
  C1 (1979) and (1985)	1	2885.6	2885.6	  2.1232	 0.0388	  9952	0.0745	
  C2 (1979,1985) and (2019)	1	25970	 25970	  23.225	 0.0001	  9941	0.0001	
Zone**	1	19138	 19138	  18.267	 0.0001	  9956	0.0001	
Year x Zone*	2	6622.1	  3311	  3.1602	 0.0001	  9914	0.0002	
   C1 x Zone	1	1605.1	1605.1	   1.181	 0.3465	  9939	0.3289	
   C2 x Zone	1	5016.9	5016.9	  4.4868	 0.0001	  9936	0.0003	
Res	32	33527	1047.7	        	       	      	      	
Total	37	86894							


Details of the expected mean squares (EMS) for the model
Source	EMS
Ye	1*V(Res) + 11.579*S(Ye)
   C1	1*V(Res) + 8.8889*S(C1)
   C2	1*V(Res) + 18.947*S(C2)
Zo	1*V(Res) + 16.364*S(Zo)
Ye x Zo	1*V(Res) + 5.7895*S(Ye x Zo)
   C1xZo	1*V(Res) + 4.4444*S(C1 x Zo)
   C2xZo	1*V(Res) + 9.4737*S(C2 x Zo)
Res	1*V(Res)

Construction of Pseudo-F ratio(s) from mean squares
Source	Numerator	Denominator	Num.df	Den.df
Ye	1*Ye	1*Res	     2	    32
   C1	1*C1	1*Res	     1	    14
   C2	1*C2	1*Res	     1	    34
Zo	1*Zo	1*Res	     1	    32
Ye x Zo	1*Ye x Zo	1*Res	     2	    32
   C1x Zo	1*C1 x Zo	1*Res	     1	    14
   C2x Zo	1*C2 x Zo	1*Res	     1	    34

Estimates of components of variation
Source	Estimate	Sq.root
S(Ye)	  	  1155.5	 33.99
   S(C1)	   171.7	 13.11
   S(C2)	  1311.6	 36.22
S(Zo)	           1105.5	 33.25
S(YexZo)	   390.9	 19.77
   S(C1xZo)	    55.4	 7.440
   S(C2xZo)	   411.5	 20.29
V(Res)	  1047.7	 32.37
PAIR-WISE TESTS (Term 'Year x Zone' for pairs of levels of factor 'Zone')
Within level '1979' of factor 'Year'
	      	       	Unique	      
Groups	     t	P(perm)	 perms	 P(MC)
RF, CG	2.9509	 0.0067	   126	 0.001
Average Similarity between/within groups
	   RF	    CG
RF	40.977      
CG	21.49	64.12

Within level '1985' of factor 'Year'
	      	       	Unique	      
Groups	     t	P(perm)	 perms	 P(MC)
RF, CG	2.1159	 0.0293	    35	 0.02
Average Similarity between/within groups
	    RF	    CG
RF	51.182	      
CG	25.607	43.257

Within level '2019' of factor 'Year'
	      	       	Unique	      
Groups	     t	P(perm)	 perms	 P(MC)
RF, CG	2.9311	 0.0002	  9430	0.0004
Average Similarity between/within groups
	    RF	    CG
RF	66.878	      
CG	48.32	 56.87

C.	Pair-wise comparisons of Distance-based test for homogeneity of multivariate dispersions (PERMDISP) on Bray-Curtis Similarity matrix of transformed square-root matrix abundance data. RF: Reef front or accretionary zone; CG: Coral hard ground or non-accretionary zone in both periods of time (before the 1990s and 2019); P(perm): P-values obtained using permutations. SE: standard error of means listed by average for each group. 

PERMDISP BRAY-CURTIS PAIRWISE COMPARISONS
Groups	      t	P(perm)	
(RF_before 1990, CG_before 1990)	1.926	 0.1046	
(RF_before 1990, CG_2019)	1.970	 0.0599	
(RF_before 1990, RF_2019)	0.076	 0.9504	
(CG_before 1990, CG_2019)	6.217	 0.0001	
(CG_before 1990, RF_2019)	2.404	   0.04	
(CG_2019, RF_2019)	2.318	 0.0379	

MEANS AND STANDARD ERRORS
Group	Size	Average	    SE	
CG2019	  10	 19.504	 1.456	
RF2019	  10	 27.661	3.2042	
RF_before_90	   9	 28.067	4.2987	
CG_before_90	   9	 37.792	2.6509	
