# Supplementary material for: The role of geomorphic zonation in long-term changes in coral-community structure on a Caribbean fringing reef
Source: PeerJ. 2020 Oct 22;8:e10103. doi: 10.7717/peerj.10103 (PMC7585725; doi:10.7717/peerj.10103)
Supplement: Supplemental Information 6 — Principal Component Analysis (PCO) derived from the Jaccard matrix constructed using a fourth root transformed matrix of standardized data of presence/absence of scleractinian coral species in two sampling zones at Punta Maroma reef seascape before 1990s and in 2019: a frontal zone of a fringing reef (RF) and a coral-ground (CG) zone. Vectors visualize, through Pearson correlation coefficient, the potential monotonic relationship between the species accounting for 70% of total abundances and ordination axes a PCO. [file peerj-08-10103-s006.pdf]

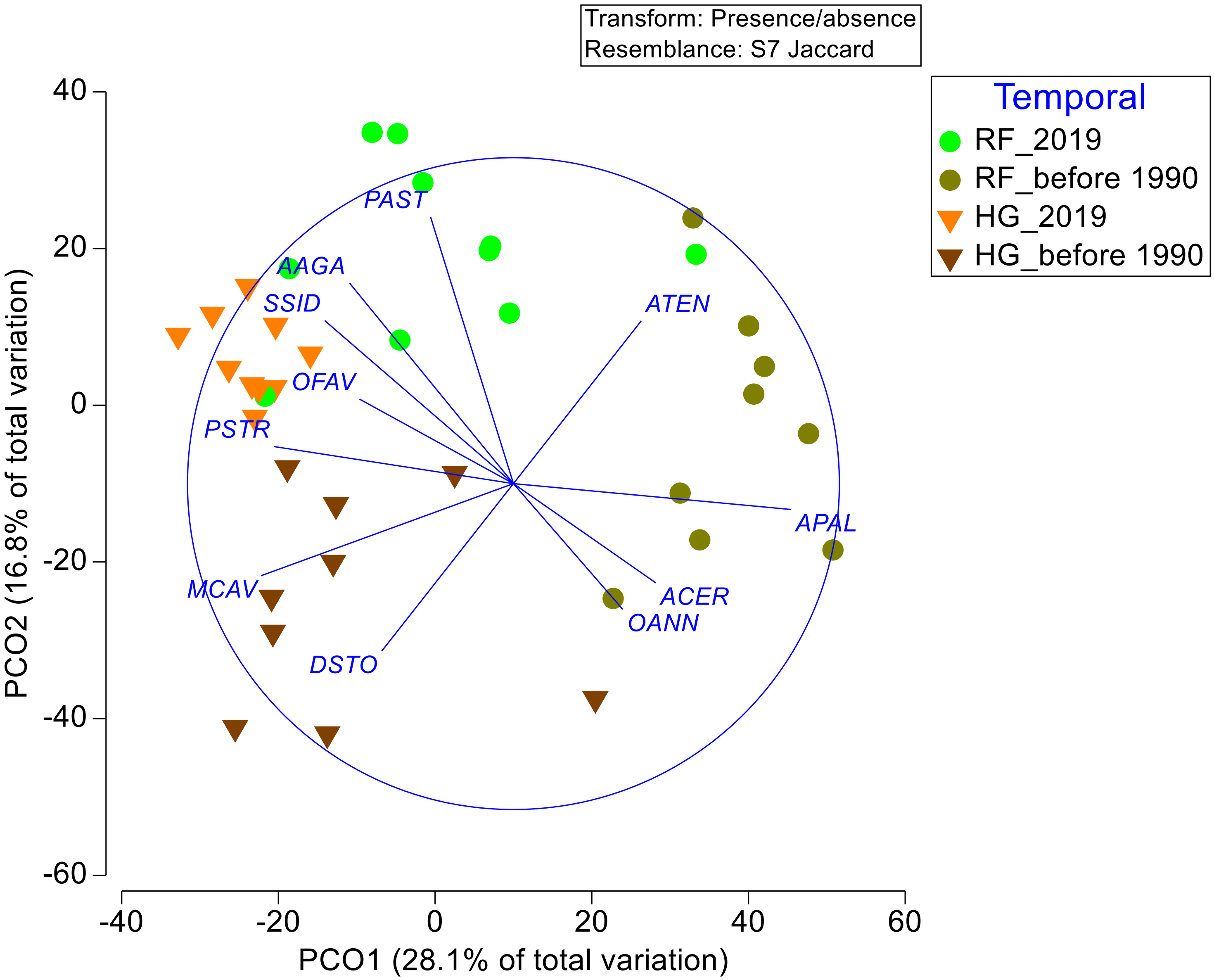

Principal Component Analysis (PCO) derived from the Jaccard matrix constructed using a fourth root transformed matrix of standardized data of presence/absence of scleractinian coral species in two sampling zones at Punta Maroma reef seascape before 1990s and in 2019: a frontal zone of a fringing reef (RF) and a coral-ground (CG) zone. Vectors visualize, through Pearson correlation coefficient, the potential monotonic relationship between the species accounting for 70% of total abundances and ordination axes a PCO. *AAGA: Ag. agaricites, ATEN: Ag. tenuifolia, ACER: Ac. cervicornis, APAL: Ac. palmata, DSTO: D. stokesii, MCAV: M. cavernosa, OANN: Orbicella spp. complex, PAST: Po. astreoides, PSTR: Ps. strigosa, SSID: S. siderea*
